# PANasta Trial; Cattell Warren versus Blumgart techniques of panreatico-jejunostomy following pancreato-duodenectomy: Study protocol for a randomized controlled trial

**DOI:** 10.1186/s13063-015-1144-9

**Published:** 2016-01-15

**Authors:** Christopher M. Halloran, Kellie Platt, Abbie Gerard, Fotis Polydoros, Derek A. O’Reilly, Dhanwant Gomez, Andrew Smith, John P. Neoptolemos, Zahir Soonwalla, Mark Taylor, Jane M. Blazeby, Paula Ghaneh

**Affiliations:** National Institutes of Health Research Liverpool Pancreas Biomedical Research Unit and Clinical Directorate of General Surgery, Royal Liverpool and Broadgreen University Hospitals NHS Trust and the University of Liverpool, Liverpool, L69 3GA UK; Cancer Research UK Liverpool Cancer Trials Unit, University of Liverpool, Block C Waterhouse Building, 1-3 Brownlow Street, Liverpool, L69 3GL UK; Department of Surgery, Manchester Royal Infirmary, Oxford Rd, Manchester, M13 9WL UK; Queen’s Medical Center, Derby Road, Nottingham, NG7 2UH UK; Department of Pancreatic Surgery, Abdominal Medicine and Surgery CSU, St James’s University Hospital, 3rd Floor Bexley Wing, Leeds, LS9 7TF UK; Churchill Hospital, Oxford University Hospitals NHS Trust, Headington, Oxford, OX3 7LJ UK; Mater Hospital, Belfast Health and Social care Trust, Crumlin Rd, Belfast, BT12 6AB UK; Bristol Center for Surgical Research, School of Social and Community Medicine, University of Bristol, BS8 2PS and University Hospitals Bristol NHS Foundation Trust, Bristol, BS2 8HW UK; Department of Molecular and Clinical Cancer Medicine, Institute of Translational Medicine, University of Liverpool, The Duncan Building, Daulby Street, Liverpool, L69 3GA UK

**Keywords:** PANasta trial, Pancreas, Cancer, Pancreato-duodenectomy, Anastomosis, Pancreatico-jejunostomy, Blumgart, Cattell-Warren

## Abstract

**Background:**

Failure of the pancreatic remnant anastomosis to heal following pancreato-duodenectomy is a major cause of significant and life-threatening complications, notably a post-operative pancreatic fistula. Recently, non-randomized trials have shown superiority of a most intuitive anastomosis (Blumgart technique), which involves both a duct-to-mucosa and a full-thickness pancreatic “U” stitch, in effect a mattress stitch, over a standard duct-mucosa technique (Cattell-Warren). The aim of this study is to examine if these findings remain within a randomized setting.

**Methods/Design:**

The PANasta trial is a randomized, double-blinded multi-center study, whose primary aim is to assess whether a Blumgart pancreatic anastomosis (trial intervention) is superior to a Cattell-Warren pancreatic anastomosis (control intervention), in terms of pancreatic fistula rates. Patients with suspected malignancy of the pancreatic head, in whom a pancreato-duodenectomy is recommended, would be recruited from several UK specialist regional centers. The hypothesis to be tested is that a Blumgart anastomosis will reduce fistula rate from 20 to 10 %. Subjects will be stratified by research site, pancreatic consistency and diameter of pancreatic duct; giving a sample size of 253 per group. The primary outcome measure is fistula rate at the pancreatico-jejunostomy. Secondary outcome measures are: entry into adjuvant therapy, mortality, surgical complications, non-surgical complications, hospital stay, cancer-specific quality of life and health economic assessments. Enrolled patients will undergo pancreatic resection and be randomized immediately prior to pancreatic reconstruction. The operation note will only record “anastomosis constructed as per PANasta trial randomization,” thus the other members of the trial team and patient are blinded. An inbuilt internal pilot study will assess the ability to randomize patients, while the construction of an operative manual and review of operative photographs will maintain standardization of techniques.

**Discussion:**

The PANasta trial will be the first multi-center randomized controlled trial (RCT) comparing two types of duct-to-mucosa pancreatic anastomosis with surgical quality assurance.

**Trial registration:**

ISRCTN52263879. Date of registration 15 January 2015.

**Electronic supplementary material:**

The online version of this article (doi:10.1186/s13063-015-1144-9) contains supplementary material, which is available to authorized users.

## Background

### Background and rationale for the study

Pancreato-duodenectomy as a procedure is 100 years old [1–3]. The “Whipple” procedure, as it became known, was refined and standardized during the 1940s to treat head of pancreas cancer [3, 4]. Preservation of the pylorus is now accepted [5–10]. Hence, the current “standard” resection preserves the pylorus with en-bloc resection of the pancreatic head to the right of the portal vein, the extra-hepatic bile duct, the gall bladder, the duodenum and the proximal jejunum. Despite centralization of pancreatic services lowering mortality to below 5 %, morbidity still remains high, often around 50 % for all causes [11, 12]. Failure of the pancreatic anastomosis to heal is a common culprit, with the medical literature quoting a pancreatic leak/fistula rate of between 2 % and in excess of 20 %, [13]. This wide range of leak/fistula rate was due partly to a lack of a consensus definition [12]; however, recently this has been proposed [13] and is beginning to gain support and be quoted in the medical literature. Health economic studies of pancreatic resection from the USA have reported an average length of stay of just over 20 days with an approximate cost of US$1000 per day [14–17]. Edge et al. [14], showed a strong association between complications, length of hospital stay and cost. Resection without complication on average had a hospital stay of 13 days and cost US$30,000. Stay and cost increases proportionally with complications, such that a minor complication increased stay to 18 days (US$43,000) and a major complication to 32 days (US$90,000). Data to 2005 suggest hospital stay is similar but that cost has increased by 63 % [18].

**Fig. 1:**
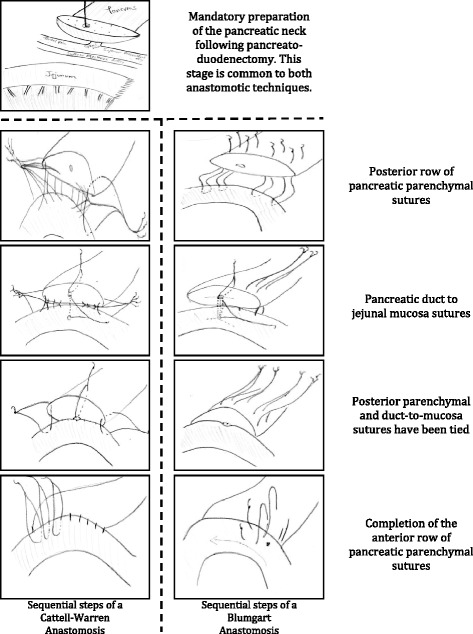
Schematic of the stages of pancreatic anastomosis construction

There are a number of techniques of pancreatic remnant reconstruction. Stump closure is a poor option as there are excessive rates of pancreatic fistula, pancreatitis and post-operative exocrine failure [19]. Advocates of pancreato-gastrostomy (PG) quote the superior nature of this technique over that of a jejunal loop, though from a poor literature base [20, 21] with meta-analyses showing conflicting results as studies are combined and both standardized and non-standardized definitions of post-operative pancreatic fistulae (POPF) are used [22, 23]. Anastomosis between the pancreatic stump and the jejunum is regarded as the “standard” technique by many pancreatic surgeons. There are two main ways to perform this: invagination of the pancreatic stump into the jejunum (pancrea*to*-jejunostomy) or anastomosis of the pancreatic duct to the jejunal mucosa (pancrea*tico*-jejunostomy), with covering parenchymal sutures. To date there is a lack of well-designed and well-conducted randomized [24–27] and non-randomized [28–32] studies, specifically looking at these techniques, which have sufficient homogeneity to be compared in meta-analyses. Design issues include the use of non-standardized definitions of POPF, a lack of surgical quality assurance, discontinuous time frames, no stratification for pancreatic consistency or diameter of the pancreatic duct, lack of consideration of the surgical learning curve and irregular use of octreotide and pancreatic duct stents across the groups. Leslie Blumgart (Memorial Sloan Kettering, New York City, NY, USA) has devised a most intuitive anastomosis to use in the setting as it involves both duct-to-mucosa stitches and a full-thickness pancreatic “U” stitch, in effect a mattress stitch. This method of placing the pancreatic parenchymal stitches has significant mechanical advantages over standard techniques as it in effect uses the jejunal mucosa as a pledgelet to minimize sheering forces of the stitches (see Fig. 1).

The rationale for this trial is that an improved method of pancreatic anastomosis should reduce POPF, decrease all complications, hospital stay, cost, improve patient-reported outcomes and promote enhanced recovery programs. Moreover, fewer POPF should enable more patients to take advantage of adjuvant therapy with a possible impact on longer-term survival.

### Preliminary data

Since the description of the “Blumgart” anastomosis, initial results show advantages [33, 34]. Moreover, Kleespies et al. [35] compared a standard Cattell-Warren pancreatico-jejunostomy with a Blumgart anastomosis (BA) technique; this reduced pancreatic leak rate from 13 to 4 % (*p* = 0.03) and overall complications from 31 to 15 % (*p* = 0.015) in favoUr of the BA. Although this study was *not* randomized and was single center, it suggests the usefulness of this technique.

### Objective

To compare the effectiveness of the BA pancreatico-jejunostomy construction versus standard treatment, that is the Cattell-Warren anastomosis (CWA), for patients undergoing an elective pancreatico-duodenectomy in terms of rates of pancreatic fistula.

### Trial design

**Fig. 2:**
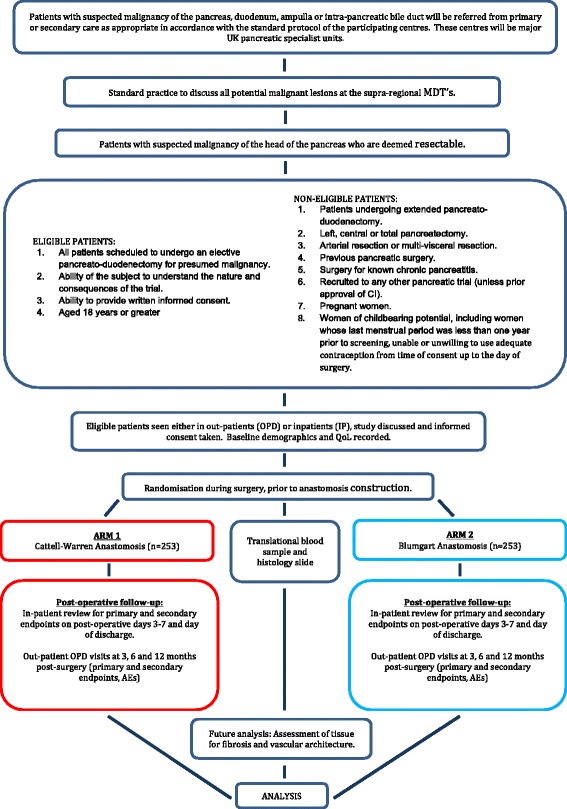
Flow diagram for the PANasta trial

The PANasta trial is a phase III multi-center study with an internal pilot phase with blinding of patients and outcome assessors (Fig. 2).

## Methods/Design

### Study setting

Screening and identification of eligible patients will take place at the research site’s pancreatic multi-disciplinary team (MDT) meeting; all such sites are supra-regional referral centers for suspected pancreatic malignancy. All patients with suspected pancreatic malignancy would undergo standard evaluation: contrast enhanced multi-detector computed tomography (CT) scan ± endoluminal ultrasound (EUS), which will be discussed at the MDT. Patients recommended for resection on the basis of a high likelihood of a malignant lesion will be contacted and provided with a participant information sheet. Informed consent will be obtained by an investigator who should comply with applicable regulatory requirements and should adhere to Good Clinical Practice (GCP) and to the ethical principles that have their origin in the Declaration of Helsinki.

It is not necessary for patients to have a histological diagnosis of malignancy before surgery provided that the MDT has confirmed that the lesion is of sufficient concern to require resection [36].

### Eligibility criteria

#### Inclusion criteria

Patients undergoing an elective pancreato-duodenectomy for presumed malignancy; ability of the subject to understand the nature or consequences of the trial; ability to provide written informed consent and be aged 18 years or older.

#### Exclusion criteria

Patients undergoing extended pancreato-duodenectomy; left, central or total pancreatectomy; arterial resection or multi-visceral resection; previous pancreatic resection; surgery for known chronic pancreatitis; recruitment to any other pancreatic resection trial (at the discretion of the chief investigator/trial team); women of childbearing potential, including women whose last menstrual period was less than 1 year prior to screening; unable or unwilling to use adequate contraception from time of consent up to the day of surgery.

#### Research site criteria

All sites and participating surgeons have expertise in operating upon pancreatic malignancy at high volumes, have confirmed experience with both methods of anastomosis and are willing to randomize patients between the two treatment groups.

### Interventions

#### Trial intervention

Reconstruction of the pancreatic remnant following pancreato-duodenectomy (either Kausch-Whipple or pylorus-preserving) using a “Blumgart” method of pancreatico-jejunostomy.

#### Control intervention

Reconstruction of the pancreatic remnant following pancreato-duodenectomy (either Kausch-Whipple or pylorus-preserving) using a “Cattell-Warren” method of pancreatico-jejunostomy.

Both techniques are regularly used, reported in the medical literature and represent standard methods of reconstruction of the pancreatic remnant. The standardization of the operative techniques will be ensured by using modified methods developed with the MRC ConDuCT-II Trials Methodology Hub [37, 38]:

1. Consensus meetings – a pre-pilot study consensus has been reached regarding the essentials of each anastomosis and the likely key steps, the post-operative management of drains, pancreatic duct stents, the use of octreotide and the timing of operative photographs. This information will be developed into a pilot-phase operative manual and tested in the main phase of the trial.
2. Operative manual – following the feasibility/pilot phase the operative manual will be reviewed and, where applicable, adapted in accordance with the information gained. A finalized operative manual for each anastomosis will be formulated and contain steps that are (a) mandatory to the construction of a safe anastomosis; (b) prohibited for the construction of a safe anastomosis, and (c) flexible steps where the operating surgeon can choose a method (please see Additional file 1).
3. Operative photographs – digital operative photographs will be taken at the key steps during anastomosis construction: preparation of the pancreatic neck prior to anastomosis; insertion of the parenchymal and duct stitches prior to knot-tying and the completed anastomosis. Photographs will be reviewed by the chief investigator and a second reviewer to confirm quality and consistency across sites.

All patients will receive an initial dose of 100 μg of octreotide before surgery. Octreotide should then be administered at 100 μg three times a day subcutaneously on post-operative days 1 to 7. Surgical drains (of any description) must be employed and should be left in place for a minimum of 3 days after surgery. If on post-operative day 3, the drain amylase is normal (i.e., less than ×3 the upper amylase limit for that institution), the drain may be removed (under the direction of the operating surgeon). The use of a pancreatic duct stent is mandatory in all patients. Acquisition of, and upload of operative photographs, as well as other mandatory steps, are recorded in the case report forms (CRFs). This is a surgical trial comparing two different methods of reconstructing the pancreatic remnant following pancreatic head resection. All usual medications and standard of medical care are accepted. There is no requirement to record any medications that are being taken by the patient.

### Outcome measures

The primary outcome measure is leak/fistula rate at the pancreatico-jejunostomy, as measured by presence/content of drain exudate and amylase content analysis within the fluid as per the International Study Group definition: see Table 1 [13]. Secondary outcome measures are: entry into adjuvant therapy/clinical trials of adjuvant therapy; mortality rate; delayed gastric empting [39]; rate of wound infections [40]; rate of pulmonary infection; rate of post-operative fluid collection; operation time; rate of intra- and post-operative bleeding [41]; rate of re-operation; rate of venous thrombo-embolism; hospital stay; generic quality of life and health economic assessments. Patients will complete the cancer- specific questionnaire, the European Organization for Research and Treatment of Cancer (EORTC) QLQ-C30, themselves whereever possible at baseline and in follow-up clinics. Postal assessments may be made as appropriate. Complications [42] that do not have agreed international standards for reporting will be assessed by the Dindo and Clavien classification [43].

**Table 1:** Post-operative pancreatic fistula: an International Study Group of Pancreatic Surgery (ISGPF) definition [14]

Grade A	Grade B	Grade C
Without clinical impact	Clinically relevant	Clinical stability may be borderline
Oral nutrition	Partial/total parental/enteral nutrition	Treatment in an intensive care unit in many cases
No antibiotics	Peripancreatic fluid collection possible	Total parental/enteral nutrition
No somatostatin analogues	Abdominal pain, fever, and/or leukocytosis possible	Intravenous antibiotics and somatostatin analogues necessary
No peripancreatic fluid collection	Antibiotics and somatostatin analogues may be necessary	Worrisome peripancreatic fluid collection that requires percutaneous drainage
No delay in hospital discharge	Delay in hospital discharge or readmission may be required.	Extended hospital stay
		Often associated complications and post-operative mortality possible

### Timeline and recruitment

Tertiary regional centers with proven track records will recruit into this trial. All local investigators have confirmed their expertise in both anastomotic techniques and are willing to randomize with clinical equipoise. Recruitment will run over 3 years with a 1-year follow-up. The schedule of trial interventions is shown in Table 2. An internal pilot study will run within the first 6 months. This will run in two centers initially from the day of the trial opening and will inform whether to continue or not dependent upon actual numbers undertaking enrollment as opposed to numbers of patients who would be eligible. Following on from the pilot phase other centers will open.

**Table 2:** Schedule of treatments. X demarcates the trial intervention

Visit	Screening	Visit1 (enrolment)	Visit 2 (Randomisation)	Post-operative days 4 and 6	Post operative days 3, 5 and 7	Visit 3 (discharge)	Visit 4 (3m FU)	Visit 5 (6m FU)	Visit 6 (12m FU)	End of Study
	To assess eligibility only (no data to be collected until consent)	Within 4 weeks of surgery	Day of surgery (Day 0)	4 and 6 days post-surgery	3, 5 and 7 days post-surgery	Day of discharge	3 months post surgery (±4 weeks)	6 months post surgery (±4 weeks)	12 months post surgery (±4 weeks)	12 months post-surgery (or earlier due to other cause)
Assessments / Procedures										
Written Informed Form		X^1^								
Assessment of eligibility criteria	X	X								
Suspected date of diagnosis		X								
Demographics (height, weight, etc.)		X				X^2^	X^2^	X^2^	X^2^	
Smoking and alcohol status		X								
Review of Medical History (including symptoms and relevant tests)		X								
Family Medical History		X								
Pregnancy Test		X								
Pancreatic Endocrine Insufficiency status		X			X	X	X	X	X	
Diabetic status		X				X	X	X	X	
Adverse events					X	X	X	X	X	
Octreotide review^3^			X	X	X (Day 3 & 5)					
Surgical drain review			X	X	X	X	X			
Randomisation			X							
CA19-9			X							
Full blood count^4^			X		X (Day 5 ±2 days)	X (Day of discharge −2 days)				
Serum Biochemistry^5^			X		X (Day 5 ±2 days)	X (Day of discharge −2 days)				
Clotting screen^6^			X		X (Day 5 ±2 days)	X (Day of discharge −2 days)				
Blood Sample for translational study^7^			X		X (Day 5 + 2days)					
Histological sample for translational study^8^			X							
Surgical Intervention			X							
Details of surgery			X							
Take Operative photographs			X							
Upload Operative Photographs^9^			X							
Operation time			X							
Intra and post-operative bleeding assessment			X		X	X				
Post- operative fluid collection				X	X	X	X			
Survival status^10^					X	X	X	X	X	X
Delayed gastric emptying assessment					X	X				
Re-operation review					X	X	X	X	X	
Pulmonary infection assessment						X	X			
Surgical site infections assessment					X	X	X	X	X	
Venous thrombo-embolism assessment					X	X	X	X	X	
Fluid collection review (to assess POPF)				X	X	X	X			
Length of initial hospital stay						X				
Re-admission review							X	X	X	
Adjuvant therapy review							X	X	X	
QoL^11^		X				X	X	X	X	
Reason for end of study										X

1. Patient consent does not need to be within 4 weeks of surgery

2. At discharge and follow up weight only to be recorded

3. Initial dose of Octreotide (100ug) to be administered on the evening before surgery (if applicable) then 100ug 3 times daily on the day of surgery and post-operative days 1 to 6

4. FBC (haemoglobin, platelets, absolute neutrophil count, white blood cell count, eosinophils, basophils, lymphocytes, monocytes) to be done pre-operatively either the evening before or morning of surgery and post-operatively on day 5. A window of **±2** days applies to day 5 FBC only and a −2 day window applies at day of discharge

5. Serum biochemistry (sodium, potassium, calcium, urea, creatinine, eGFR, random glucose, albumin, bilirubin, alk.phosphatase, total protein, AST or ALT, GGT and CRP) to be done pre-operatively either the evening before or morning of surgery and post-operatively on day 5. A window of **±2** days applies to day 5 serum chemistry only and a −**2** day window applied at day of discharge

6. Clotting screen (PT and APTT) to be done pre-operatively either on the evening before or morning of surgery and post-operatively on day 5. A window of **±2** days applies to day 5 clotting screen only and a −**2** day window applies at day of discharge

7. Translational blood samples (10ml EDTA tube and 8.5ml SST tube) to be taken pre-operatively either on the evening before or morning of surgery and on post-operative day 5. A window of **+2** days applies to the day 5 translational blood samples

8. Diagnostic H&E slide of the pancreatic neck transection margin will be requested for all patients along with a histological report by the LCTU on a 6 monthly basis. Slides will be held for the duration of the trial and returned at the end

9. Operative photographs should be uploaded on to the LCTU portal immediately after surgery

10. Death due to any cause must be reported by completing an End of Study Form

11. EORTC QLQ-C30 and EQ-5D and EQ-VAS to be completed

### Sample size

The primary outcome is the binary measure of pancreatic leak or no leak. Current experience suggests the present rate of leak is 20 % in the standard treatment; a reduction to a 10 % rate in the trial treatment is of clinical importance. A sample size of 208 patients per group would allow a two-sided, two-sample test for binomial proportions to detect a difference in proportions of 10 % (10 % versus 20 %) with power of 80 % and significance level of 0.05. The sample size calculation was based on an O’Brien-Fleming two-stage design with binding futility boundaries (SAS PROC SEQDESIGN version 9.3). Non-compliance would occur in the event of unresectable disease at laparotomy (estimated at 15 %) with a further assumed loss to follow-up of 3 %, giving an overall sample size of 253 per group (506 in total).

### Randomization

Randomization will be undertaken intra-operatively by the surgeon following pancreatic head excision and just prior to pancreatic remnant reconstruction. Hence, the surgeon will only randomize the participant if they feel at complete equipoise and are happy to perform either anastomosis. Randomization will take place via a password-protected web-based tool called the Treatment Allocation Randomization System (TARDIS) developed, maintained and controlled by Liverpool Cancer Trials Unit’s IT Department. Patients will be randomized to one of the treatment groups in a 1:1 ratio, with the following stratification factors: (1) research site, (2) pancreatic texture: soft versus normal/hard, and (3) pancreatic duct diameter: 3 mm or >3 mm.

### Blinding

Patients and all team members except for the surgeons will be blinded to the intervention. In order to maintain the blinding for the patient and other site staff, the surgeon will not write which anastomosis has been performed in the surgical notes but rather this will be recorded in the notes as “anastomosis constructed as per PANasta trial randomization.” Serious adverse events (SAEs) will be assessed blinded, and only if deemed necessary would un-blinding occur, i.e., if subjects require life-saving surgery in a district hospital. This, however, would be a rare event. Adverse event (AE) and SAE reporting would follow as soon as is practical. This will not have any impact on the endpoint assessment of the patient.

### Data management

This study is run from the Liverpool Cancer Trials Unit (LCTU). The study CRF is the primary data collection instrument for the study. CRF pages will be available for sites to download from the LCTU portal: http://www.lctu.org.uk.

Individual participant medical information obtained as a result of this study is considered confidential and disclosure to third parties is prohibited. CRFs will be labelled with patient initials and unique trial screening and/or trial number. Intra-operative photographs will be transferred to the LCTU and will be identifiable by unique trial number only. Histological slides will be transferred to the Department of Pathology at the Royal Liverpool University Hospital and will be identifiable by unique trial number only. Blood samples will be transferred to the University of Liverpool GCLP laboratory and will be identifiable by unique trial number only. Consent forms sent to the LCTU as part of the randomization process may contain patient identifiers for the purpose of monitoring as described in the trial risk assessment. Such information will be stored in secure, locked cabinets.

### Withdrawals

If voluntary withdrawal occurs, the patient will be asked to allow continuation of scheduled evaluations, complete an end-of-study evaluation, and be given appropriate care under medical supervision until any AE symptoms resolve or the subject’s condition becomes stable. Subjects are free to leave the trial at any point without the need to give reasons for their decisions. If voluntary withdrawal occurs prior to the surgical intervention the patient will not be randomized and no further trial data will be collected on that patient. Patients may wish to withdraw from the trial following the surgical intervention. In such cases no further data should be collected but data up to this time can be included in the trial if anonymized. Patients may also wish to remove all data and/or any samples collected up to the point of withdrawal from the trial analysis. In these cases an End of Study Form along with the reason for withdrawal will be submitted to the LCTU. However, safety data that have been sent to regulatory authorities prior to that point (e.g., Annual Safety Reports to Research Ethics Committee) will not be withdrawn. For patients moving from the area, every effort should be made for the patient to be followed-up at another participating trial center and for this trial center to take over responsibility for the patient or for follow-up via the GP.

### Statistical methodology

Formal interim statistical monitoring of the accumulating data will be performed at regular intervals and reviewed by an Independent Data Monitoring and Safety Committee (ISDMC). In addition, the design allows for an interim analysis with possible early stopping after results for 208 patients are available. At this stage, based on an O’Brien-Fleming two-stage design, if the standardized *Z* value lies in the interval (−0.698, 0.698) then the trial is stopped for acceptance of the null hypothesis of equal proportions. If the standardized *Z* value is less than −2.736 or greater than 2.736 then the trial is stopped accepting one proportion is significantly less than the other. Otherwise the trial continues to the final stage where the test for the standardized *Z* value will have acceptance region for the null hypothesis of (−1.934, 1.934). Values outside this region will indicate significantly different proportions. (SAS PROC SEQDESIGN version 9.3 was used for the design.)

Early stopping rule: the first 6 months of the study will be run as a feasibility/pilot study with a decision, whether to continue or not, made at that point. Two sites will be opened initially for this feasibility/pilot phase. The decision whether to continue or not will be based on the number found to be eligible across the two sites, the number enrolled, the number randomized and the number who took up the randomization. If the number enrolled at 6 months is less than 40 patients, then a decision will be made as to whether to continue the trial or not. If the recruitment rate is acceptable then the other sites will be opened, with an expected number of patients at 12 months of 137 across all sites.

The trial will be analyzed and reported following the Consolidated Standards of Reporting Trials (CONSORT) guidelines [44, 45]. All statistical analyses will be on an intention-to-treat basis. Missing data, which are anticipated to mainly affect the quality of life outcome measure, will be handled by considering the robustness of the complete case analysis to sensitivity analyses using different imputation assumptions informed by data collected on reasons for missing data. Continuous variables will be summarized by descriptive statistics (mean, standard deviation, minimum, median and maximum) and frequency tables will be provided for categorical data. The two-sided, two-sample test for binomial proportions will be used to test for a difference in the probabilities of leak/fistula occurrence between the two arms. Sensitivity analysis will be performed using the continuity-corrected Cochran-Mantel-Haenszel (CMH) test calculated over the recruiting centers. Logistic regression will be used to investigate the variation in these probabilities by center. The secondary endpoints will be analyzed using summary statistics, CMH tests, logistic regression, analysis of variance and multi-variate methods as appropriate. As the two procedures are standard practice, a formal analysis of the primary endpoint only will be undertaken when half the patients have been recruited. Quality of life measurements will be assessed over time and comparisons made between treatment groups using longitudinal analysis with appropriate recognition for informative dropout.

### Trial oversight committees

#### Trial Management Group (TMG)

This comprises the chief investigator, other lead investigators (clinical and non-clinical) and members of the LCTU. The TMG will be responsible for the day-to-day running and management of the trial and will meet approximately three times a year.

#### Trial Steering Committee (TSC)

The Trial Steering Committee will consist of the TMG plus independent members, two co-investigators and a patient representative. The TSC will provide overall supervision for the trial and provide advice through its independent chairman. The ultimate decision for the continuation of the trial lies with the TSC.

#### Independent Data and Safety Monitoring Committee (IDSMC)

The Independent Data and Safety Monitoring Committee (IDSMC) will be responsible for reviewing and assessing recruitment, interim monitoring of safety and effectiveness, trial conduct and external data. The ISDMC will first convene prior to trial opening and will then define frequency of subsequent meetings (at least annually). The ISDMC will provide a recommendation to the TSC concerning the continuation of the study.

### Trial organization and monitoring

This trial is sponsored by the University of Liverpool (UoL000732) and is co-ordinated through the LCTU. This study has been funded by Cancer Research UK (CRUKE/13/019). Site monitoring is conducted to ensure protection of patients participating in the trial, trial procedures, laboratory, trial intervention administration, and data collection processes are of high quality and meet sponsor and, when appropriate, regulatory requirements. The trial will be audited periodically by the University of Liverpool and the LCTU quality assurance manager. A risk assessment in accordance with the MRC/DH/MHRA Project on Risk-adapted Approaches to the Management of Clinic Trials of Investigational Medicinal Products (http://www.mhra.gov.uk/home/groups/l-ctu/documents/websiteresources/con111784.pdf) has been undertaken for the PANasta trial. As this is a surgical intervention trial of two standard techniques of reconstructing the pancreatic remnant the risk assessment resulted in a trial category of Type A, and thus the trial is considered to be low-risk.

### Safety and adverse event reporting

#### Adverse event (AE)

Any untoward medical occurrence (i.e., any unfavorable or unintended sign including abnormal laboratory results), symptom or disease in a research participant to whom a medicinal-/-clinical investigation has been administered, including occurrences which are not necessarily caused by or related to that product-/investigation.

#### Serious adverse event (SAE)

Any AE is classified as serious if it:

1. Results in death
2. Is life-threatening (subject at immediate risk of death)
3. Requires in-patient hospitalization or prolongation of existing hospitalization
4. Results in persistent or significant disability or incapacity, or
5. Consists of a congenital anomaly or birth defect
6. Important medical events that may not be immediately life-threatening or result in death or hospitalization but may jeopardize the patient or may require intervention to prevent one of the other outcomes listed in the definition above should also be considered serious

All AEs that occur from time of surgery up to the point at which a patient starts adjuvant therapy should be reported. The assignment of the severity or grading should be made by the investigator who is responsible for the care of the participant. Regardless of the classification of an AE as serious or not, its severity must be assessed according to medical criteria (Table 3). The assignment of causality will be assessed according to the definitions outlined in Table 4.

**Table 3:** Definitions of severity

Definition of severity of adverse event	Grade	Description
1. Mild	Grade 1	Does not interfere with patient’s usual function (awareness of symptoms or signs, but easily tolerated (acceptable)
2. Moderate	Grade 2	Interferes to some extent with patient’s usual function (enough discomfort to interfere with usual activity (disturbing)
3. Severe	Grade 3	Interferes significantly with patient’s usual function (incapacity to work or to do usual activities (unacceptable)
4. Life-threatening	Grade 4	Results in risk of death, organ damage, or permanent disability (unacceptable)
5. Death	Grade 5	Results in death (unacceptable)

**Table 4:** Definitions of causality

Relationship	Description
None	There is no evidence of any causal relationship. N.B. an alternative cause for the AE should be given
Unlikely	There is little evidence to suggest there is a causal relationship (e.g., the event did not occur within a reasonable time after the trial procedure). There is another reasonable explanation for the event (e.g., the participant’s clinical condition, other concomitant treatment)
Possibly	There is some evidence to suggest a causal relationship (e.g., because the event occurs within a reasonable time after the trial procedure). However, the influence of other factors may have contributed to the event (e.g., chemotherapy or other concomitant treatments)
Probably	There is evidence to suggest a causal relationship and the influence of other factors is unlikely
Highly probable	There is clear evidence to suggest a causal relationship and other possible contributing factors can be ruled out

The expected events for this trial along with reporting obligations are detailed in Table 5. Expected events (primary and secondary endpoints) for the trial are exempt from SAE reporting unless they are classified as life-threatening or result in death (grades 4/5). Events that are exempt from SAE reporting should be reported in the relevant sections of the CRFs and *do not* need to be reported as an SAE. All other events that meet the criteria for serious *must* be reported as an SAE and those that do not feature as expected should be classified as *unexpected*. An AE where the causal relationship to the study procedure is assessed by the investigator as “possible,” “probable,” or “highly probable,” is graded as serious and *unexpected* is subject to expedited reporting to the Main Research Ethics Committee (MREC). This is the responsibility of the LCTU.

**Table 5:** Expected and unexpected events for serious adverse event (SAE) reporting

Expectedness	Event	Grade 3 and below		Grade 4 and above	
		Report as SAE	Subject to expedite reporting	Report as SAE	Subject to expedite reporting
Expected	Pancreatic fistula (graded as A, B or C)	No	No	Yes	No
	Delayed gastric emptying (graded as A, B, or C)	No	No	Yes	No
	Wound infections	No	No	Yes	No
	Pulmonary Infection	No	No	Yes	No
	Post-operative fluid collection	No	No	Yes	No
	Intra- and post-operative bleeding	No	No	Yes	No
	Re-operation	No	No	Yes	No
	Venous thromboembolism	No	No	Yes	No
	Interventional drainage procedures	No	No	Yes	No
	Extended hospital stay due to delayed surgery	No	No	Yes	No
	Surgical complication related hospital stay	No	No	Yes	No
	New post-operative pancreatic exocrine and or endocrine failure	Yes	No	Yes	No
Unexpected	Any other serious event deemed to be unrelated to the surgical intervention	Yes	No	Yes	No
	Any other serious event deemed to be related to the surgical intervention	Yes	Yes	Yes	Yes

### Ethical considerations

This study has been submitted to the National Research Ethics Service, Committee North West – Greater Manchester South (Ref. 14/NW/1393) and received a favorable opinion on 22 December 2014.

### Protocol amendments

Version 1 (22 September 2014), version 2 (6 October 2014), version 3 (10 October 2014), version 4 (5 January 2015) and version 5 (22 July 2015).

### Translational research

In addition to the routine blood assessments, translational blood samples will be taken either the evening before or the morning of surgery and then again at post-operative day 5. Sample collection kits will be sent to all participating sites and the samples collected and stored to GCP standard in Liverpool. These samples will be used at a future date to evaluate individual cytokine levels correlated to outcome of each anastomosis. In addition the hematoxylin and eosin slide of the transected pancreatic neck (which is standard pathological reporting) will be assessed for amount of fibrosis and vascular architecture and again correlated to each anastomosis.

### Indemnity

PANasta is sponsored by the University of Liverpool and co-ordinated by the LCTU in the University of Liverpool. The University of Liverpool does not hold insurance against claims for compensation for injury caused by participation in a clinical trial and cannot offer any indemnity. However, in terms of liability, NHS trust and non-trust hospitals have a duty of care to patients treated, whether or not the patient is taking part in a clinical trial, and they are legally liable for the negligent acts and omission of their employees. Compensation is, therefore, available in the event of clinical negligence being proven.

### Dissemination

The results from different centers will be analyzed together and published as soon as possible. The TMG will form the basis of the Writing Committee and will advise on the nature of publications.

## Discussion

Pancreato-duodenectomy for a tumor in the head of the pancreas is now a standardized procedure. Although centralization of services has driven mortality down, the morbidity, if honestly reported, coupled with sensitive definitions and active, prospective ascertainment of complications, is around 50 %. Although some complications are modest, failure of the pancreatic remnant anastomosis to heal is a major cause for a number of significant and life- threatening complications. In addition, pancreatic anastomotic breakdown causing a POPF is a cause of increased length of stay and cost to the healthcare provider, notwithstanding misery for the patient and the potential of delayed adjuvant chemotherapy, until the fistula is healed.

There are many options available to surgeons when considering pancreatic anastomosis; however, this has muddied the waters rather than provided clear evidence. Thus, to date there are neither randomized nor non-randomized trials of any of the techniques [20–33]; whether comparing reconstruction to the stomach versus a jejunal loop, or duct to mucosa versus invagination of jejunal limbs, to adequately construct meta-analyses that inform the medical literature. Those that are published, are littered with homogeneity and bias relating to: non- standardized definition of POPF, non-standardized technique, no attempt at internal quality control, discontinuous time frames, no stratification of soft/hard pancreatic consistency or diameter of pancreatic duct, unfamiliarity with technique at some centers and irregular use of octreotide and pancreatic duct stents.

**Fig. 3:**
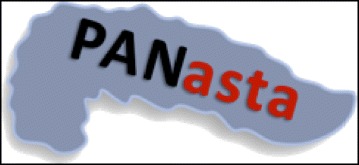
PANasta logo

The PANasta trial (logo Fig. 3) is unique in that it will compare two different methods of pancreatic duct-to-mucosa pancreatico-jejunostomy: Blumgart versus Cattell-Warren anastomoses. Hence, the PANasta trial aims to identify an improved method of pancreatic anastomosis which should reduce POPF, decrease all complications, hospital stay, cost and promote enhanced recovery programs. Moreover, fewer POPF should enable more patients to take advantage of adjuvant therapy.

This study is ambitiously intending to randomize over 500 patients undergoing pancreatic surgery. These numbers are powered to detect a 10 % absolute point reduction in the primary endpoint of POPF – which is a relevant difference in surgical trials. Included is a generous dropout rate and account is also taken of patients who are not randomized if unresectable disease is encountered at laparotomy. Furthermore, randomization will be undertaken following resection, just prior to pancreatic anastomosis; therefore, maximising surgeon equipoise. One of the prime reasons that the current medical literature base on pancreatic anastomosis is so poor is the lack of operative standardization not only between individuals but also between centers. In order to diminish this as much as is possible a number of safeguards are built into this study: notably incorporation of a feasibility pilot study within the first 6 months; a pre-pilot consensus meeting; formulation of an operative manual and operative photographs.

The internal pilot will run in two centers initially and will evaluate in step-wise fashion: (1) the number of patients eligible at each site; (2) the number of patients consented; (3) the number of patients randomized; and finally (4) the number of patients who took up the randomization. This will inform us of the real figures for stopping this trial for futility in recruitment. In addition, an interim analysis will be undertaken once 208 patients have been randomized.

The pre-pilot consensus meeting was attended by the research site principal investigators (PIs), who agreed that the use of octreotide, pancreatic duct stents and post-operative drains were to be used in *all* patients. The steps of each anastomosis were discussed and agreed. The adherence to these steps will be reviewed at the end of the internal pilot phase and the manual and protocol modified as necessary.

An operative manual (Additional file 1) has been constructed and will be followed during the pilot phase of this study. The investigators will meet following the pilot study, prior to the main study opening to re-evaluate which steps are *mandatory*, *prohibited* or *flexible* to the construction of a safe anastomosis. Furthermore, during both the pilot and full trial operative photographs will be taken of the three key phases of each anastomosis. These will be continuously monitored and any recurrent defects will be reported to the ISDMC. Both the operative manual and operative photographs will form the quality control and standardization of the trial beyond the pilot phase into the full trial.

In conclusion, the PANasta trial will be the first multi-center randomized controlled trial (RCT) to compare two methods of pancreatico-jejunostomy (Blumgart versus Cattell-Warren) to assess the impact on pancreatic fistula and leak. This study is, therefore, planned to provide important evidence to inform health policy and clinical decision-making relevant to patients undergoing pancreatico-duodenectomy for presumed cancer of the pancreas.

## Trial status

Open and recruiting: “green light” 23 April 2015; first patient enrolled 29 April 2015 and first patient randomized 5 May 2015.

## Additional file

Additional file 1:
**PANasta operative manual.** (PDF 520 kb)

## Abbreviations

AE: adverse event
BA: Blumgart anastomosis
CMH: Cochran-Mantel-Haenszel
CRF: case report form
CWA: Cattell-Warren anastomosis
DH: Department of Health
EUS: endoscopic ultrasound
GCP: Good Clinical Practice
GCLP: Good Clinical Laboratory Practice
ISDMC: Independent Data and Safety Monitoring Committee
LCTU: Liverpool Cancer Trials Unit
MDT: multi-disciplinary team
MHRA: Medicines and Healthcare Products Regulatory Agency
MRC: Medical Research Society
MREC: Main Research Ethics Committee
PG: pancreatico-gastrostomy
POPF: post-operative pancreatic fistula
SAE: severe adverse event
TMG: Trial Management Group
TSC: Trial Steering Committee
