# Supplementary material for: PANasta Trial; Cattell Warren versus Blumgart techniques of panreatico-jejunostomy following pancreato-duodenectomy: Study protocol for a randomized controlled trial
Source: Trials. 2016 Jan 15;17:30. doi: 10.1186/s13063-015-1144-9 (PMC4714471; doi:10.1186/s13063-015-1144-9)
Supplement: Additional file 1: — PANasta operative manual. (PDF 520 kb) [file 13063_2015_1144_MOESM1_ESM.pdf]

Operative Manual

PANasta Trial
Cattell Warren versus Blumgart techniques of panreatico-jejunostomy following pancreato-duodenectomy – a double blinded multi centred trial

Table of Contents

1. Outline for Use of the Operative Manual 3
   1.1 Role in the Pilot phase……………………………………….....3
   1.2 Role beyond the pilot phase……………………………......3
2. Surgical Procedures... 4
   2.1 Preparation of the pancreatic neck prior to reconstruction of the pancreatic remnant 4
   2.2 Cattell-Warren Anastomosis........ 5
      2.2.1 Parenchymal Sutures 5
      2.2.2 Pancreatic Duct-to-Mucosa of Jejunum Sutures 6
      2.2.3 Completion of the Anastomosis 7
   2.3 Blumgart Anastomosis........ 8
      2.3.1 Parenchymal Sutures 8
      2.3.2 Pancreatic Duct-to-Mucosa of Jejunum Sutures 9
      2.3.3 Completion of the Anastomosis 10
3. Other Mandatory Steps... 11
   3.1 Octreotide 11
   3.2 Drains 11
4. Other Equivocal Steps 11
   4.1 Biliary Reconstruction........ 11
   4.2 Gastric Reconstruction........ 11
   4.3 Route of Reconstruction........ 11
5. Appendix: Local Site Photographic Policy 12

1. Outline for the use of this Manual

This manual has been constructed to provide guidance and ongoing quality assurance such that the construction of either a “Cattell-Warren” anastomosis or a “Blumgart Anastomosis” following pancreatoduodencetomy can be performed in a standardised fashion.

1.1 Role in the Pilot phase

It is acknowledged that surgeon preference will play a role in anastomosis construction, hence as this manual develops a number of steps which will either be classified as **MANDATORY**, **EQUIVOCAL** or **PROHIBITED**, will develop.

The two pilot sites (Royal Liverpool University Hospital and Central Manchester Trust), will work in close participation, with the use of operative photographs and video of each surgeons variation on technique. At completion of the pilot the steps will be formalised prior to opening to the other national sites.

1.2 Role beyond the pilot phase

It is expected that constant reference to the manual will be undertaken to ensure continued standardisation of techniques beyond the pilot phase of the study.

Standardisation of technique will be assessed during the study period by taking photographs of the three key stages of the anastomosis:

PHOTOGRAPH 1: Preparation of the mobilised pancreatic neck prior to anastomosis.

PHOTOGRAPH 2: Insertion of the parenchymal and duct stitches prior to ting the knots.

PHOTOGRAPH 3: The completed anastomoses.

2. Surgical Procedures

2.1 Preparation of the pancreatic neck prior to reconstruction of the pancreatic remnant.

MANDATORY STEP FOR BOTH ANASTOMOSES

Following removal of the surgical specimen and prior to reconstruction of the anastomosis the neck of the pancreas requires preparation.

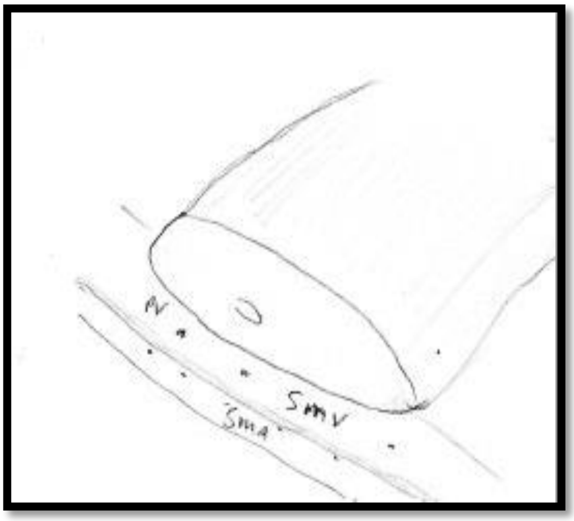

This step is **MANDATORY** for both the Cattell-Warren and the Blumgart anastomosis. The extent of mobilisation will depend on how far the neck was divided to the left of the spleno-portal confluence.

The neck of the pancreas is gently mobilised from the underlying venous confluence by judicious use of dissection, diathermy and ligation of small tributaries. This should be for a minimum of 15mm from the cut edge of the pancreas. Haemostasis should be secured.

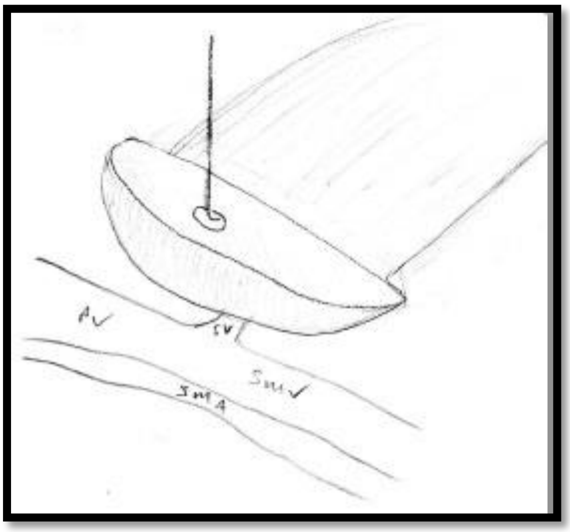

PHOTOGRAPH (1) REQUIRED:
Preparation of the mobilised pancreatic neck prior to anastomosis

2.2 Cattell-Warren Anastomosis (CWA)

The operating surgeon is able to use their preferred suture material / suture caliber (**EQUIVOCAL**) for both the parenchymal and duct-to-mucosa elements of this anastomosis

2.2.1 Parenchymal Sutures

This anastomosis is begun by inserting a suture at the corner approximately 10mm from both the side and the cut edge of the pancreas, then picking up a serosal bite of jejunum approximately 5mm in length.

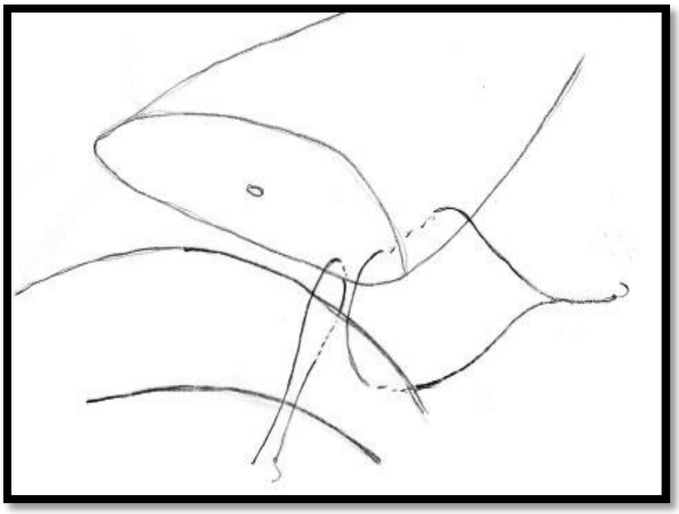

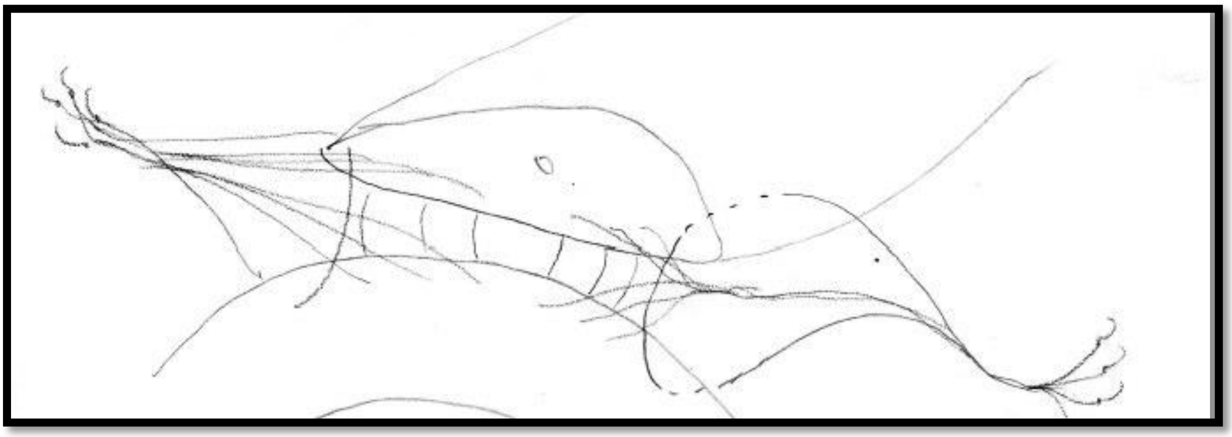

Further sutures are placed medial to the corner stitch again approximately 10mm from the cut edge and with a 5mm bite of jejunum, spaced evenly along the posterior border of the pancreas. Care is taken not to obstruct the pancreatic duct. All parenchymal sutures are placed before duct sutures are placed.

The pancreatic duct is rarely central, such that more sutures may be required on one side than the other (**EQUIVOCAL**).

2.2.2 Pancreatic Duct-to-Mucosa of Jejunum Sutures

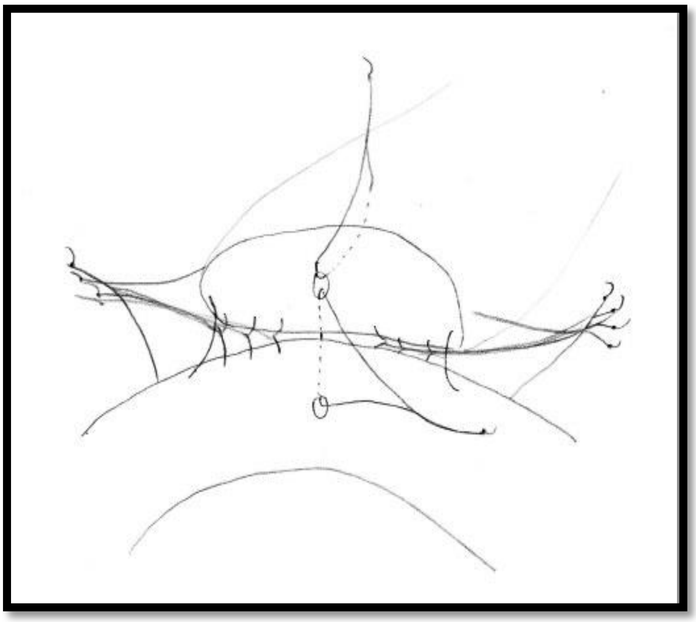

The jejunal loop is transposed next to the pancreas and a full thickness hole created by diathermy approx. the same diameter as the pancreatic duct, adjacent to the pancreatic duct. Sutures of appropriate calibre are inserted from the pancreatic duct to the mucosa of the jejunum.

The size of bite will depend on the thickness of the pancreas (**EQUIVOCAL**). Whether the suture passes fully through the pancreatic parenchyma or whether it is brought through the cut edge of the pancreas is of surgeon preference (**EQUIVOCAL**).

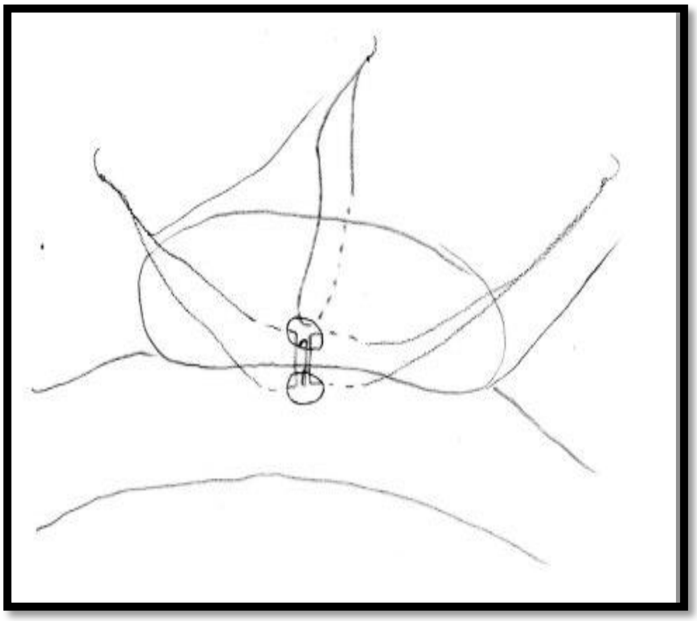

Further pancreatic duct to jejunal mucosal sutures are inserted.

There is no minimum or maximum number of sutures,but they should be placed equidistant around the duct (**EQUIVOCAL**).

PHOTOGRAPH (2) REQUIRED:
Insertion of the parenchymal and duct stitches prior to tying the knots.

2.2.3 Completion of the Anastomosis

The ***duct*** sutures are held taught while the parenchymal sutures are sequentially tied with secure knots starting medially and working laterally; first on one side then on the other.

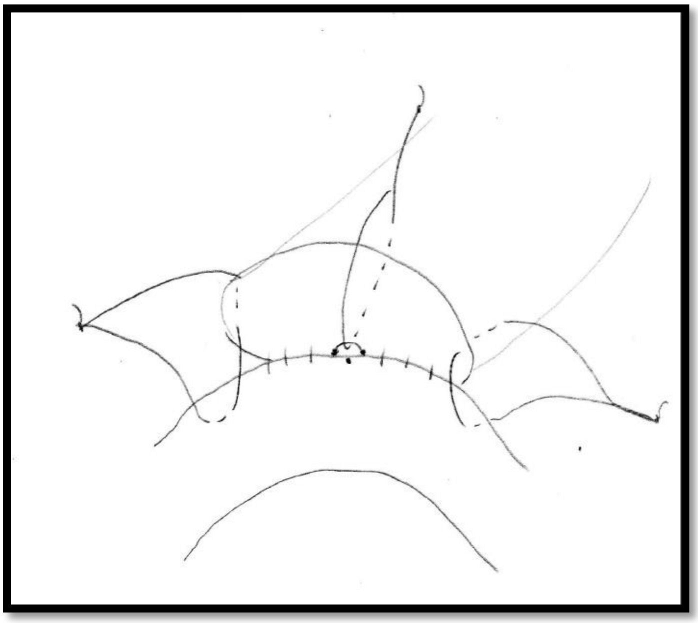

Once these have been tied and the excess suture removed the duct-to-mucosa sutures are sequentially tied.

The use of a pancreatic duct stent is **MANDATORY**, and needs to be confirmed on the surgery CRF. The stent may be placed during the anastomosis or at the end prior to ting the last duct stitch.

Finally an anterior row of parenchymal to jejunal sutures are inserted. The size of bite will depend on the thickness of the pancreas (**EQUIVOCAL**). A sufficient number of sutures are required to oppose the pancreas and the jejunual loop along its entire length.

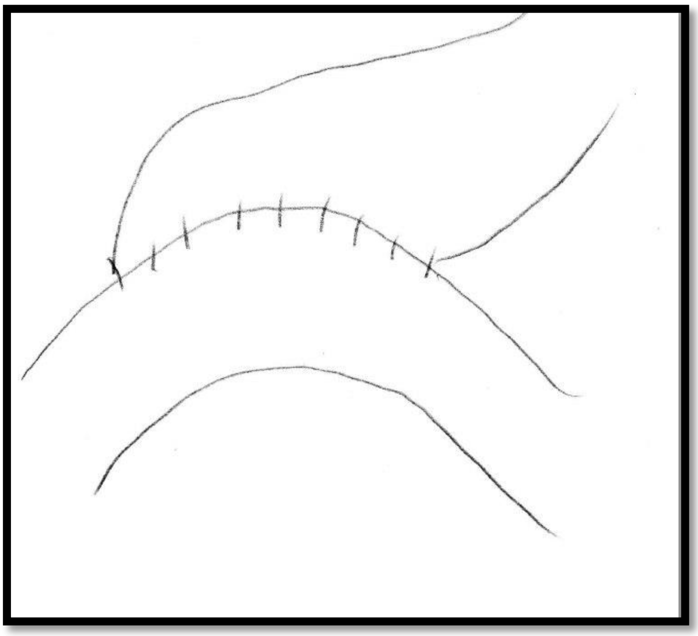

PHOTOGRAPH (3) REQUIRED:
The completed anastomosis.

2.3 Blumgart Anastomosis (BA)

The operating surgeon is able to use their preferred suture material / suture caliber (**EQUIVOCAL**) for both the parenchymal and duct-to-mucosa elements of this anastomosis.

2.3.1 Parenchymal Sutures

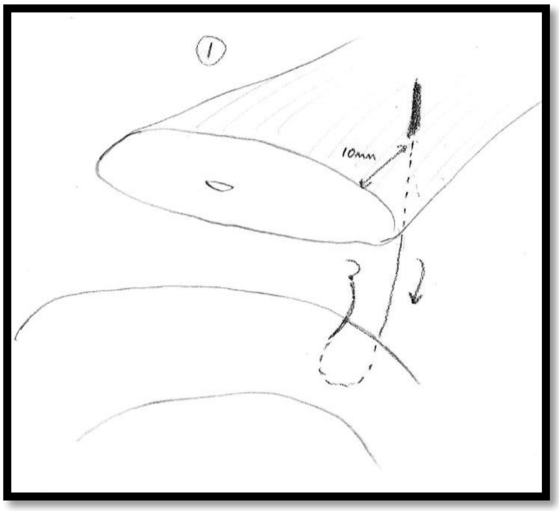

The anastomosis is begun by inserting an appropriate suture full thickness through the pancreatic parenchyma, at one edge approximately 10mm form the cut edge of the pancreas. A bite of jejunal serosa approximately 5mm is then taken with the same suture.

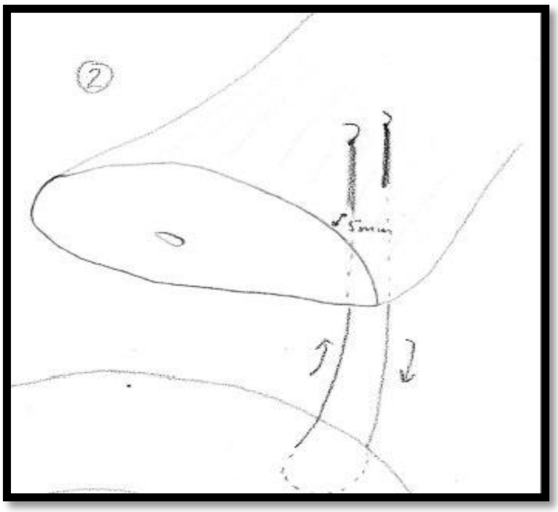

The same suture is then doubled back and reinser ted through the pancreat i c parenchyma, opposite the initial bite but now approximately 5mm from the cut edge of the pancreas.

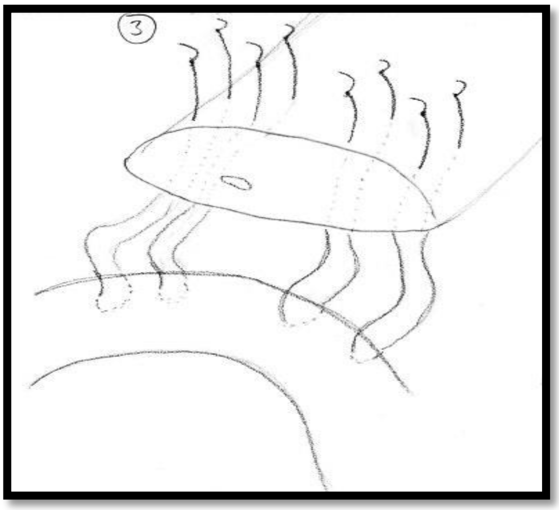

This is repeated along the pancreas either side of the pancreatic duct. The pancreatic duct is rarely central, such that more sutures may be required on one side than the other (EQUIVOCAL).

The maximum number of sutures is 4, 2 each side of the duct (**MANDATORY**). The minimum number of sutures is 3, 2 one side of the duct and 1 the other (**MANDATORY**).

2.3.2 Pancreatic Duct-to-Mucosa of Jejunum Sutures

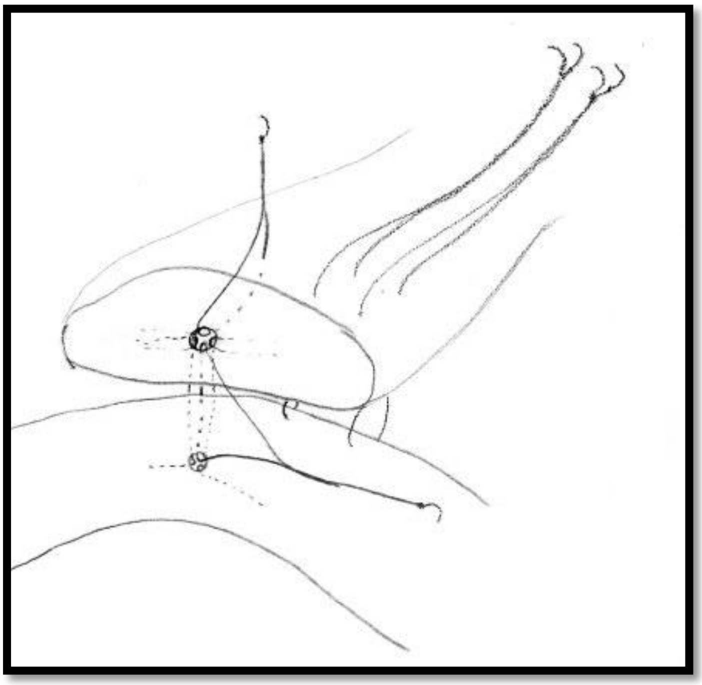

The jejunal loop is transposed next to the pancreas and a full thickness hole created by diathermy approx. the same diameter as the pancreatic duct, adjacent to the pancreatic duct. Sutures of appropriate caliber are inserted from the pancreatic duct to the mucosa of the jejunum.

The size of bite will depend on the thickness of the pancreas (**EQUIVOCAL**). Whether the suture passes fully through the pancreatic parenchyma or whether it is brought through the cut edge of the pancreas is of surgeon preference (**EQUIVOCAL**).

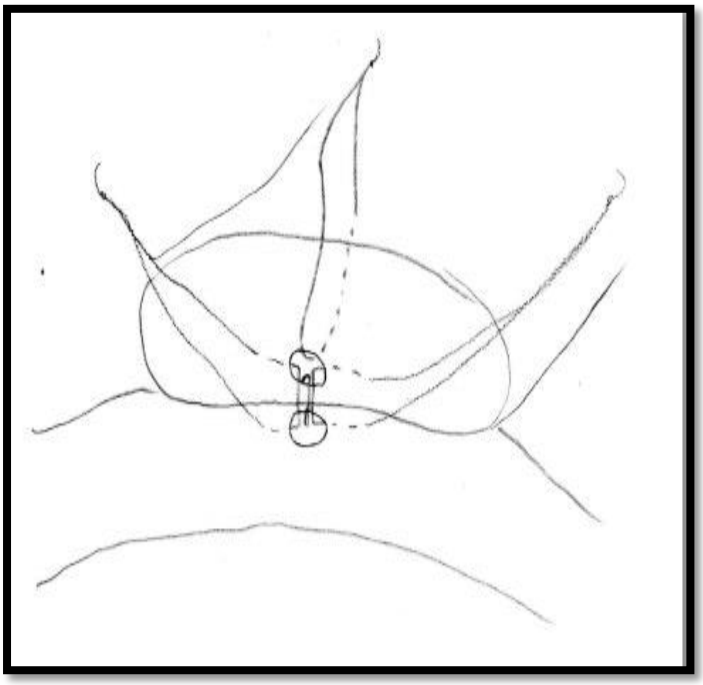

Further pancreatic duct to jejunal mucosal sutures are inserted.

There is no minimum or maximum number of sutures, but they should be placed equidistant around the duct (**EQUIVOCAL**).

PHOTOGRAPH (2) REQUIRED
Insertion of the parenchymal and duct stitches prior to tying the knots.

2.3.3 Completing the Anastomosis

The ***parenchymal*** sutures are held taught, thus pulling the posterior layer of the jejunum around the pancreas, while the duct sutures are sequentially tied with secure knots.

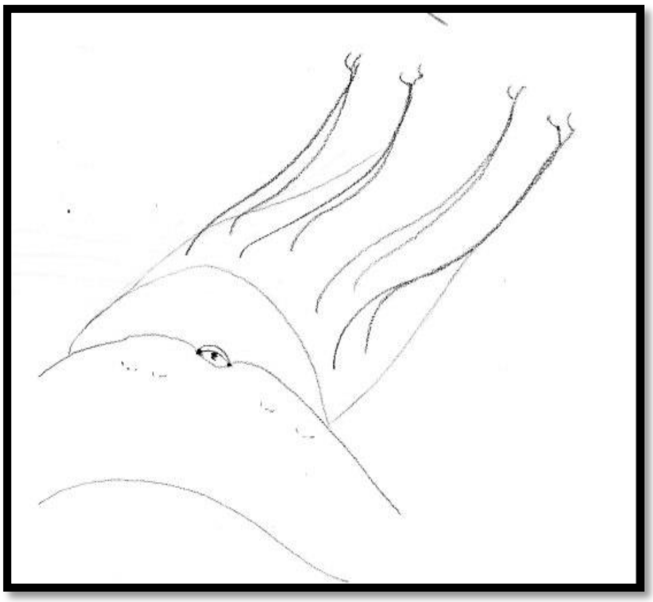

The use of a pancreatic duct stent is **MANDATORY**, and needs to be confirmed on the surgery CRF. The stent may be placed during the anastomosis or at the end prior to ting the last duct stitch.

The parenchymal sutures are then passed back through the anterior level of the jejunal loop, thus pulling the anterior layer of the bowel over the top of the pancreas.

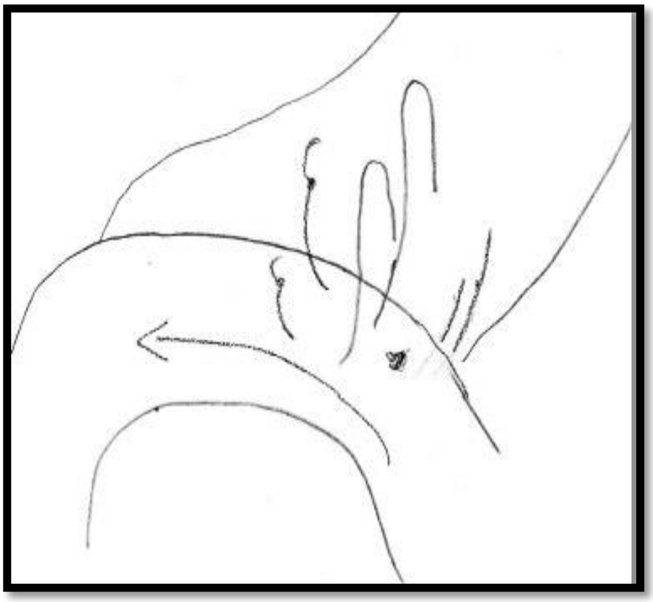

This can either be undertaken as 1 or as 2 bites (as shown opposite), depending on the surgeon preference (**EQUIVOCAL**).

The knots are secured.

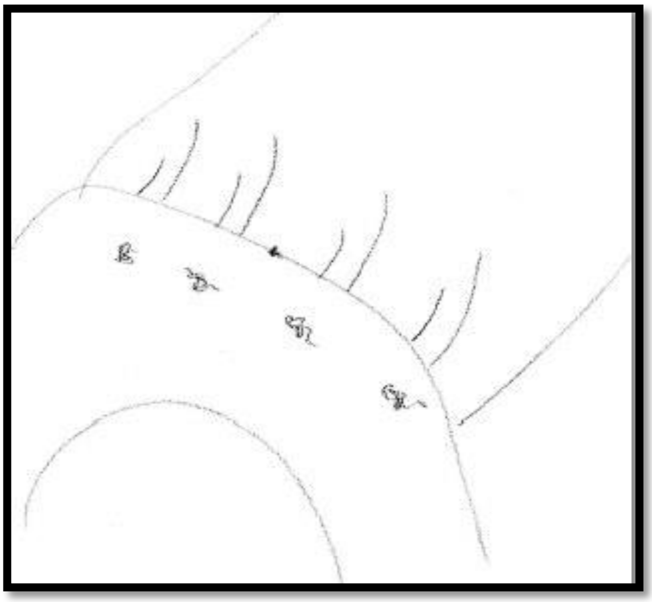

PHOTOGRAPH (3) REQUIRED
The completed anastomosis.

3. Other Mandatory Steps

3.1 Octreotide
The use of octreotide has been discussed and by consensus it was agreed that all cases should receive 100ug, subcutaneously, three times daily for seven days. This should commence prior to surgery.

3.2 Drains
The use of drains (of any description) must be employed and should be left for a minimum of three days. If the drain amylase is recorded as normal on post-operative day three, the drains may be removed (under the direction of the operating surgeon).

Other centres will keep drains for longer and sequentially shorten. Any drainage of fluid from the drains or drain sites will be analysed for amylase content on post-operative days 3, 4, 5, 6 and 7. If a drain is still in-situ at discharge, amylase will be measured.

4. Other Equivocal Steps

The PANasta study is exploring differences only in the pancreatic remnant anastomosis.

4.1 Biliary Reconstruction
This can be performed according to the preference of the operating surgeon, but a description should be entered into the CRF.

4.2 Gastric Reconstruction
This can be performed according to the preference of the operating surgeon, but a description should be entered into the CRF.

4.3 Route of Reconstruction
This can be performed according to the preference of the operating

5. Appendix: Local Site Photographic Policy

Note: If applicable, please attach local policy.
